# Bringing Forth Within: Enhabiting at the Intersection Between Enaction and Ecological Psychology

**DOI:** 10.3389/fpsyg.2020.01348

**Published:** 2020-08-14

**Authors:** Mark M. James

**Affiliations:** School of Computer Science, University College Dublin, Dublin, Ireland

**Keywords:** enaction, ecological psychology, sense-making, umwelt, enhabiting, Simondon, individuation

## Abstract

Baggs and Chemero (2018) propose that certain tensions between enaction and ecological psychology arise due different interpretations about what is meant by the “environment.” In the enactive approach the emphasis is on the umwelt, which describes the environment as the “meaningful, lived surroundings of a given individual.” The ecological approach, on the other hand, emphasises what they refer to as the habitat “the environment as a set of resources for a typical, or ideal, member of a species.” By making this distinction, these authors claim they are able to retain the best of both the ecological and the enactive approaches. Herein I propose an account of the individuation of habits that straddles this distinction, what I call a compatabilist account. This is done in two parts. The first part teases out a host of compatibilities that exist between the enactive account as developed by Di Paolo et al. (2017) and the skilled intentionality framework as developed by Bruineberg and Rietveld (2014) and Rietveld and Kiverstein (2014). In part two these compatibilities are brought together with the philosophy of Gilbert Simondon to develop the notion of enhabiting. Enhabiting describes a set of ongoing processes by which an umwelt emerges from and is reproduced within the relationship between an embodied subject and their habitat. Thus, enhabiting points toward a point of intersection between enaction and ecological psychology. To enhabit is bring forth (to enact), within (to inhabit).

## Epigraph

Still, what happens if a breakdown is so severe that the agent is not, so to speak, “caught” within any particular activity or genre? There is likely at this stage a hiatus of deep disorientation, of simultaneous partial abandonment and retention of the old frame of significance. We may find ourselves still involved in some of the previous schemes, only that they do not seem to make much sense now. In fact, until the situation is resolved and a new microworld emerges, we are world-less.

(Di Paolo et al., 2017, p. 167)

## Introduction

Convergences between enaction and ecological psychology are “many and strong,” according to Di Paolo (2016a, p. 327). Both reject explanatory strategies understanding cognition as consisting in the manipulation of content-involving representations. Both emphasize contextuality over reductionism, foreground particularity and process, and stress the constitutive role of body–environment relationships in the development of cognition (Szokolszky et al., 2019). Given such convergences, some suggest that they are ripe for integration (e.g., Kiverstein and Rietveld, 2018). An integrated perspective might offer “a systematized and consistent post-cognitivist approach to cognition” (Heras-Escribano, 2019, p. 1). Elsewhere, there is less certainty unification possible, and, as Di Paolo (2016a) also puts it, enactivists and ecological psychologists “stare at each other across an uncanny valley” (p. 327).

Segundo-Ortin et al. (2019) contend that when offering an anti-representationalist alternative, ecological psychology can get along well without enaction. Chemero (2012) contends that the theory of autopoiesis informing many enactive perspectives is “a troublingly idealistic theory” (p. 54). And Fultot et al. (2016) argue that enaction retains an implicit representationalism, lacks principled grounding, embeds an animal–environment dualism, and is purely constructivist position despite protestations otherwise.

Enactivism, although often relying on ecological psychology for empirical support, tends to be skeptical of the realism entailed by traditional approaches and implications about a “pre-given” environment, and dissatisfied by the apparent inability to provide any substantive account of value or the individuality of action. Reflecting these concerns in a comparison between approaches, Varela et al. (1991, p. 204) write, “Gibsonians treat perception in largely optical (albeit ecological) terms and so attempt to build up the theory of perception almost entirely from the environment. Our approach, however, proceeds by specifying the sensorimotor patterns that enable action to be perceptually guided, and so we build up the theory of perception from the structural coupling of the animal.”

This paper will not attempt to synthesize the approaches into a “systematized and consistent” whole. Building upon some recent work by Baggs and Chemero (2018, 2019), a *compatibilist* approach is advocated, i.e., a plurality of complementary frameworks^1^. The compatibilist approach centers on intelligibility rather than systematicity and consistency. It can address phenomena that concern embodied cognitive scientists more completely and more sensitively to the “externalities” of theoretical application^2^. Traditionally, the various approaches have different emphases and often provide consistent accounts of the phenomena they interrogate. There are, however, some phenomena that demand contributions from both approaches. The emergence of habits is one explored here.

Baggs and Chemero (2018, 2019) argue that the confusion between approaches can be circumvented by acknowledging their different explanatory strategies. Each has a different starting point. The ecological approach has an ontological strategy, focused on characterizing the “environmental” structure that affords adaptive possibilities. The enactive approach has an epistemological strategy, focused on how a history of acting structures one’s “environment” so it calls forth existing skills (Baggs and Chemero, 2018). Such differences are revealed in how they employ the notion of affordance. There are three primary camps.

The first, the more traditional ecological perspective, is the *affordances as dispositions* camp (e.g., Turvey et al., 1981; Turvey, 1992; Wilson, 2018a). Here, affordances are lawlike and enduring environmental “dispositions.” They are enduring even in the absence of any who would make use of them and thus capable of applying selection pressures. As Wilson (2016) writes, affordances have “to be “out there” and made of things that light can bounce off.” Given the lawful relationship between the structure in light and the structure in that which it reflects off, it can carry directly meaningful “information about” the available affordances. As the organism moves about its environment, it “picks up” this information and can thus act on the available affordances. Such affordances, as dispositions of the environment, are paired with dispositions in the organism, so-called *effectivities*, and whenever the two meet, a certain course of action follows (Turvey et al., 1981).

This dispositional account has its critics. Because any individual in a species can, in theory, occupy the same point of observation relative to the surfaces around him or her, he or she is thought to have access to the same “information about.” This supposition allows Gibson (1966, p. 321) to claim that, “The basis for agreement among men exists in the available stimulus information.” However, by focusing on the environmental structure there to be *found*, it de-emphasizes learning in shaping what any particular individual actually finds. In Baggs and Chemero’s (2018, p. 6) language, it “fails to account for the fact that a newspaper that is written in a particular language affords reading only for a certain subset of the world’s population, namely the set of people that are literate in that language.” A corollary of this is that if information is directly meaningful and available in the structure of the light (sound etc.), then there is nothing to be learned (Adolph and Kretch, 2015). And finally, this account struggles to make sense of within-individual variability. It “leaves obscure,” as Baggs and Chemero (2018, p. 8) put it, “the conditions under which a given affordance is actualised.” If affordances are dispositional properties of environments acted upon in the presence of a related “effectivity,” any time affordance and effectivity are present to one another, the affordance should be acted upon (Chemero, 2009). But this is quite obviously not the case.

The second position is the enactively informed *affordances as relations* camp (e.g., Chemero, 2003, 2009; Stoffregen, 2003). Here, affordances are relational entities that arise only under certain organism–environment configurations. This perspective was originally posed by Chemero (2003, 2009) to integrate insights concerning the role of environmental information with insights from enaction concerning the sources of value and the particularities of individual perception. There are prominent critiques here also. Wilson (2016) highlights the most troubling of them: it is not clear how one perceives a relation of which they are part, and any capacity for affordances to apply selection pressures is negated, for they arise with the ability but do not precede it. Consequently, learning novel relational affordances is impossible. In the relational account, “organisms co-create affordances by their causal interactions with the environment. This means that I can only create affordances using abilities I already have; so how do I learn new affordances? It can’t be by being in the presence of those new affordances, because I cannot create them yet …” (Wilson, 2018b). In other words, within the account of affordances more agreeable to a typically enactive perspective, it is difficult to account for the emergence of novel relational affordances.

A third position is the *affordances as practices* camp of the skilled intentionality framework (hereafter SIF) (e.g., Bruineberg and Rietveld, 2014; Rietveld and Kiverstein, 2014). They develop a relational account too, but expanded from the purely “material” to the “sociomaterial” (van Dijk and Rietveld, 2017, p. 6). Here, affordances are defined as “relations between aspects of the sociomaterial environment in flux and abilities available in a form of life” (van Dijk and Rietveld, 2017, p. 10). A “form of life” relates to the practices common within a given species, their “relatively stable and regular ways of doing things” (Rietveld and Kiverstein, 2014, p. 328). We are not just sensitive to the material affordances of the hammer, but its role within its larger context. Such insights were inspired most recently by Dreyfus and Hubert (1992) responding to the so-called “frame-problem.” However, they can be originally traced to Heidegger (1927/1962). Heidegger (1927/1962), for instance, spoke about comprehending the tool against a background or network of other tools and uses that gave the tool its meaning, what he called a “totality of equipment” (p. 97)^3^. van Dijk and Rietveld (2017) use the example of climbing stairs to highlight the sociomaterial nature of affordances, describing how one’s steps might reflect an awareness that people are sleeping nearby. The stairs afford not just *climbing* but, you might say, *climbing quietly so as not to wake the others in the house who are up early in the morning for work*.

The SIF makes important contributions by recognizing affordances as being contextualized by larger *fields* and *landscapes* of affordances, i.e., the sociomaterial contexts that shape action at multiple timescales. By allowing attention to be oriented by more experienced individuals, through observation or training, learners can attune to the available affordances within a form of life. This account makes room for individual variation while not defining affordances in terms of individual abilities (van Dijk and Rietveld, 2018), allowing for affordances to drive selection and accommodate learning within a form of life. However, it is not clear how without an account of affordances also tied to individual abilities radically novel practices can emerge, or how an individual within a given practice might innovate beyond the boundaries of its present configuration.

All of these accounts make important contributions to questions of learning, but also harbor limitations^4^. The dispositional account argues for the kinds of structures necessary in the environment to guide learning but underdetermines the historicity of learning and the particularities of a given organism–environment relation. The relational account, although highlighting how a history of learning determines the affordances one is likely to make use of, is closed to the emergence of novel affordances at an individual level. And the practice account, although allowing for a relational account in which affordances still apply selection pressures, seems to come up short in its ability to account for the emergence of radically novel practices or innovations. One suggestion about the nature of these shortcomings is the idea that these accounts are focused on the *what* rather than the *how* of learning. As Cariani (2016, p. 324) puts it, “Both constructivist and ecological psychology theories need to explicitly incorporate concrete processes of learning alongside what is or can be learned.” But there *are* frameworks right across the valley that speak about the *how* of learning.

On the ecological side, the *perceptual learning* of Eleanor Gibson (1969, 1994), which focused on processes of selection and differentiation of a sufficiently rich stimulus. Or the contemporary progeny of such accounts, such as the *direct perceptual learning* theory of Jacobs and Michaels (2007), which is centrally concerned with how the acting agent comes to “identify useful, complex information–environment specificities at the level of ambient energy arrays, under universal constraints captured by natural laws and local constraints given by a specific task situation” (Szokolszky et al., 2019). Here, learning is an information-guided process in which attention becomes progressively more attuned to optimally useful information (see Szokolszky et al., 2019, for discussion).

On the relational side, the most comprehensive account of learning is the *equilibration* account put forth by Di Paolo et al. (2017). Here, the focus is on how stable sensorimotor correlations evolve through the resolution of tensions in the relationship between existing structures and structures in the environment (Di Paolo et al., 2017, p. 88). Their account provides insights into the developmental dynamics that support learning and the logic for why any act of perception reflects an individual history. Consequently, they provide an answer to the question of why the agent is attuned to “some particular subset” of environmental information “that has meaning to it at this moment” (Di Paolo, 2016a, p. 327).

The “issue” with these perspectives is not the internal details of the accounts themselves, but that they maintain the limitations highlighted in the various accounts of affordances. Thus, what is introduced here is not an amendment to any of the accounts in particular, though both may be informed by it. Rather, it is intended as a framing within which conversations between these various approaches might be couched given their shared interests in making intelligible the dynamics of situated action.

Following Baggs and Chemero (2018), this starts with the recognition that the apparent incompatibilities with the above approaches result from attributing *some* reality, just different types, to each notion of affordance. The enactive approach emphasizes the structure of experience and how the world emerges in the relationship between organism and “environment.” In other words, here emphasis is placed on the *umwelt*, which describes the environment as the “meaningful, lived surroundings of a given individual” (Baggs and Chemero, 2018, p. 6). One primary value of the enactive perspective is the epistemic limits it sets, reminding us that the knower is always implicated in the known. Nevertheless, enactivists tend to conceive of their project not in the idealist terms attributed to them earlier, but as a kind of middle way, and are even committed to a basic ontological “realism” of sorts, i.e., there are some sort of mind-independent structures that we can come to know, even if coming to know “them,” we render them mind-dependent. Enactivists thus speak about the “structural coupling” of organism and environment (Varela et al., 1991). But accounts of the structure that make up the environmental side of the coupling are admittedly underwhelming.

More classically ecological approaches, given their ontological focus and their desire for an account of how structures in the environment can be a source of selection pressures, emphasize what Baggs and Chemero (2018) refer to as the *habitat*: “the environment as a set of resources for a typical, or ideal, member of a species” (p. 6). Importantly, the “habitat” does not designate physical reality writ large, but rather, the set of material relations that exist prior to and independently of any individual member of a species that could in theory impact them. “The habitat,” writes Baggs and Chemero (2018, p. 7), “is the physical world described relative to a potential actor, or set of actors.” This account speaks of a dispositional account of affordances, and its recognition is valuable for it provides the basis for an empirically grounded anti-representationalist approach to understanding perception and action, one that helps acknowledge the basic intuition that we occupy a shared world despite our individual histories.

Holding this distinction, Baggs and Chemero claim, we can retain the best of both approaches.

The affordance concept serves a different purpose depending on whether we invoke it in the habitat or in the umwelt. In the former case, affordances are dispositional properties, or persisting resources that exist across generations and exert evolutionary selection pressure. In the latter case, they are relational properties that exist for only so long as a given animal continues to live, and that change as that animal develops new skills and abilities, or loses them.

(2018, p. 8)

In the compatibilist account, the ecological perspective clarifies what might be said about the environmental side of the structural coupling, thus supporting the commitments throughout the valley to the possibilities of an account of perception and action grounded in the language of science. The enactive perspective, on the other hand, reminds us that even such a language is but a frame onto the world^5^.

The following article comes in two primary parts. Part I teases out existing tensions between certain enactive and ecological accounts, suggesting that if we maintain the distinction that Baggs and Chemero (2018) introduce, they can be understood as reflecting underlying compatibilities. There are, nowadays, enactivisms (e.g., Hutto and Myin, 2013; Villalobos and Ward, 2015; Cummins and De Jesus, 2016; Di Paolo et al., 2017) and ecological psychologies (e.g., Gibson, 1979; Chemero, 2009; Rietveld and Kiverstein, 2014; Wilson, 2018a). The focus here is on teasing out compatibilities between the sometimes called “autopoietic enactivism” associated with Di Paolo et al. (2017) (hereafter enactivism) and the skilled intentionality framework associated with Bruineberg Rietveld (2014) and Rietveld and Kiverstein (2014), primarily within ecological psychology. The primary reason for focusing on these accounts is that both already acknowledge the importance of insights from ecological and enactive perspectives, and both have some central role for the notion of autonomy and thus compatibilities are already present that can be further refined. Importantly, the SIF is something of a marginal view within the ecology of ecological psychology. The developments herein do aim to be informative within that ecology. However, given available space, discussion is limited to comparisons between the perspectives mentioned. That said, future developments will benefit from engagements with more classically articulated ecological perspectives.

Starting from a shared concern with the idea of self-maintenance, a path is woven through a host of related notions, highlighting compatibilities along the way. Firstly, the central notions of *sense-making* and *tending toward optimal grip*. From there, through related concepts concerning the abilities of agents, the timescales that organize action, the role of the “environment,” and questions around identity and normativity. Concluding this first part, it is suggested that the compatibilities highlighted can be brought into a more enduring relationship through the necessity of their mutual deployment in accounting for the individuation of novel habit structures. Within this account, this process is termed *enhabiting*. Developing the notion of enhabiting is the focus of part II. Inspired by the philosophy of Gilbert Simondon, it offers an account it offers an account of the ongoing constitution of habitual organizations at multiple timescales, through establishing interdependencies between bodily structures and structures in the habitat.

## Part I: Finding Compatibilities

A starting point for thinking about compatibilities between these two approaches is simply pointing out that both accounts are centrally concerned with processes of self-maintenance. The enactive approach continues in the tradition of Maturana and Varela (1987) and describes self-maintenance in terms of autopoiesis (or autonomy more generally). The SIF borrows from the Fristonian account (Friston, 2009, 2010) and describes self-maintenance in terms of the free energy principle (FEP). As Kirchhoff (2016) points out, comparing the originary accounts, they both “converge on … the organizational property for living systems: self-maintenance through a process of autopoiesis” (p. 8). “One can show,” Kirchhoff goes on, “that the process of autopoiesis is a process that minimizes free energy” (Kirchhoff, 2016). Given the demands of space, the extent to which this claim is true or not is not explored here (see Kirchhoff, 2016, for discussion). Rather, by contrasting the concepts typically used to describe the activities that support self-maintenance, *sense-making* and *tending toward optimal grip*, we can begin teasing out the compatibilities that will be reverent to the positive account later on.

### Sense-Making and Tending Toward Optimal Grip

*Sense-making*, within enaction, describes the activity of an adaptive autonomous body directed at its ongoing viability. In short, it describes a “bodily process of adaptive self-regulation” (Di Paolo and Thompson, 2014, p. 9). The self-production of the biochemical networks constitutive of organismic life, or *autopoiesis*, requires ongoing and periodic access to various material and energetic resources. As such, actions are appraised as better or worse according to their ability to satisfy these requirements. Consequently, the autopoietic instantiation provides a meaningful background against which activities and events are made sense of, a “natural perspective from which encounters in the world are intrinsically meaningful for the organism following the norm established by the continuing process of self-production” (Di Paolo, 2005, p. 429–430). Thus, the job of sense-making is the *maintenance* of the identity of the organic body. Recently, however, sense-making has been expanded to include not just the maintenance of autonomous biochemical identities (life), but sensorimotor identities also, in the form of habits, networks of habits (ways-of-life), and so on (Barandiaran, 2017; Di Paolo et al., 2017).

In the SIF, sense-making is replaced with the notion of *tending toward an optimal grip*. Kiverstein and Rietveld (2018) write that “We characterise … sensemaking activity in terms of the tendency toward an optimal grip on multiple affordances” (p. 156). This notion originates in the work of Merleau-Ponty (1945) and has been long championed by Dreyfus (2002).

“According to Merleau-Ponty, in absorbed, skilful coping … acting is experienced as a steady flow of skilful activity in response to one’s sense of the situation. Part of that experience is a sense that when one’s situation deviates from some optimal body–environment relationship, one’s activity takes one closer to that optimum and thereby relieves the “tension” of the deviation. One does not need to know, nor can one normally express, what that optimum is.”

(Dreyfus, 2002, p. 378)

The agent is moved to improve its grip on its environment by neutralizing tensions in the relationship, by continually negating deviations from an optimum. As Bruineberg and Rietveld (2014, p. 12) put it, “an organism self-organizes by reducing a disequilibrium in the brain–body–environment system.” As tensions manifest in our experience, they “solicit” action. There is not necessarily some explicit goal state organizing action here, “the skilled individual does not have an explicit goal in mind, but rather is solicited or invited by the field of affordances … what is at the root of skilled activity is not a set of desires or goals, but rather the ongoing modulation of coupled self-organizing dynamical systems that results in the adequate interaction of an organism with its environment” (2014, p. 3). Illustrative examples include finding the best angle for a photo, adjusting your distance in a queue, editing a text, or playing chess. Living systems are continuously striving to improve grip (Kiverstein et al., 2019). Consequently, tending toward optimality might be considered a very basic norm shaping the regulatory dynamics of the organism–environment relation that support self-maintenance. In line with the FEP account, tending toward optimal grip entails the progressive movement of the organism toward better “models” of their environment over time. Unlike closely related Bayesian constructions that focus on brain processes (e.g., Clark, 2015; Kiefer and Hohwy, 2018), this does not posit structural representational models carrying representational content. “Under the FEP, models are not explicitly encoded by physical states … states of the brain. Rather, it is the adaptive behavior of the system that implements or instantiates a generative model” a statistical “prediction” or anticipation of optimal behaviors in their particular “econiche” (Ramstead et al., 2019). The agent resonates with its environment in ways that prepare it for acting therein. They are attuned. The language of “modeling” will be troubling here for some, as decoupling it from its representationalist implications is something of a challenge (see Ramstead et al., 2019, for an insightful account that supports the use of the language of modeling in non-representationalist terms)^6^. Having introduced these central notions, we can now consider some of the tensions and compatibilities that follow.

### Habits and Abilities

Habit is a relatively recent development within enaction (Di Paolo, 2003; Barandiaran, 2008, 2017; Barandiaran and Di Paolo, 2014; Ramírez-Vizcaya and Froese, 2019; James and Loaiza, 2020). However, it is an important one, for it is said to supply a “blending category between the biological and the psychological,” and “a theoretical building block for an organicist conception of mind” (Egbert and Barandiaran, 2014, p. 2). Barandiaran has defined habits as “self-sustaining patterns of sensorimotor coordination formed when the stability of a particular mode of sensorimotor engagement is dynamically coupled with the stability of the mechanisms that generate it, and which is reinforced through repetition” (Barandiaran, 2008). Habits demonstrate forms of circular self-production analogous to other autonomous forms, such as autopoiesis. A single habit, contends Barandiaran (2017), provides “a first analogy with life and a first approximation to a sensorimotor conception of identity and normativity,” whereby “through repetition … a habit can take on a life of its own: it is both the cause and the consequence of its own enactment” (p. 13). What emerges within the habit is a minimal sense of identity, a focal point concerned with its own maintenance. Given that any habit relies on certain conditions – rate of repetition, particular sociomaterial structures, etc – boundaries of viability are enacted, stipulating certain actions as required if the habit is to be kept alive, i.e., the norms of its own self-regulation (Barandiaran, 2017). Inspired by this account, but following the constraint cycle position advocated by Loaiza et al. (2020) (included in this topic collection), an alternative definition is offered here. A habit is a self-sustaining ecobehavioral entity in which structure and operation enable each other in a closed circular fashion, relations which are reinforced, growing more autonomous, when repeated within appropriate timescales. The specifics of this definition will become apparent in later sections.

Importantly, the enactive account also moves beyond single habits to self-reinforcing, self-cohering networks of inter-regulating habits that unfold across longer timescales. When the network’s plastic interconnectedness is complex enough, sensorimotor regulations engender large-scale equilibrating tensions within the network, whereby “sensorimotor compensations … take place to maintain the capacity of the agent to keep behaving coherently” (Barandiaran, 2017, p. 14). At this point, the network’s self-conservation becomes its basic operational norm, and it enables activities that sustain its identity as such. Now, rather than the organic whole being the sole background against which sense is made, habits and networks of habits are also self-maintaining, norm-generating backgrounds shaping the sense-making of the embodied subjects that instantiate them. Any such structure, regardless of timescale of operation or domain of relevance, will be referred to as a *sense-making* frame, or a *sense frame* (hereafter SF) for short^7^. The idea that sense-making operates within a “frame” and that sense-making entails the reconstruction of such “frames” has surfaced elsewhere in the enactive literature. See Di Paolo et al. (2018, p. 36) or the Di Paolo et al. (2017, p. 167) – the epigraph of this article) for examples of this language. Although Di Paolo et al. (2017) do not elaborate on the notion, what they are pointing toward is precisely the kind of autonomous organizations considered here. Habits, bundles of habits, and autopoietic biochemical structures all constitute SFs.

As habitual ecobehavioral entities, the norms of SFs can partially decouple from the normative dimensions of the autopoietic structures upon which they lean. As such, they can even instantiate self-regulating norms that function counter to the norms of autopoiesis (Barandiaran, 2017). As Di Paolo (2009, p. 18) puts it, “the inherent regulative tendencies of sophisticated processes of identity generation are likely to sometimes enter into conflict even with basic metabolic values.” Examples include participating in extreme sports, excessive consumption of intoxicants, and so on. The behavior of the embodied subject is simultaneously motivated by the self-production of one particular identity (e.g., a way of life as a big wave surfer) while threatening another (the organic living whole) and inhibiting the expression of habits that would otherwise support it. This can result in challenging states of dissonance. Indeed, many so-called “bad habits” get their name for this reason (Ramírez-Vizcaya and Froese, 2019). Thus, one recognizes some inter-regulatory dynamics at work in the relationship between different forms of autonomy.

Where enaction speaks of skillful action as subtended by integrated networks of habits, the SIF speaks of *abilities*. Within the SIF, affordances are the relationship between features of a sociomaterial environment and abilities in a form of life (van Dijk and Rietveld, 2017, p. 10). Any individual, at any time, is embedded within a “field of affordances,” however, only some subset of the field stands out as relevant, the “field of relevant affordances.” To say that they “stand out” suggests that they are experienced as soliciting behavior (Dreyfus and Kelly, 2007; Rietveld and Kiverstein, 2014). This depends upon a bodily “action readiness” on the behalf of the skilled actor (Frijda et al., 1989) whereby within a given situation the individual is attuned, or “selectively open,” to certain features of their environment, anticipating what they are likely to encounter, and readying themselves to act so as to be responsive to the demands of the situation (van Dijk and Rietveld, 2017). The abilities of a given individual then, which have taken shape through a history of engaging in sociomaterial practices (Rietveld, 2008), are reflected in the patterns of action readiness, selective openness, and skillful response that manifest in any particular situation. Tending toward optimal grip, one is continually responding to solicitations, and thus constantly reorganizing the dynamics of the body–environment system such that the field of relevant affordances is in continuous flux.

The emphasis in this account, as previously noted, is more on the side of the environment. Given such emphasis, however, the SIF fails to account for the richness of “abilities” that the inter-regulating plastic structures captured in the enactive account suggest. Abilities are simply far too coarse-grained a notion. For instance, one does not merely have abilities or not have abilities. Rather, one has abilities and varying degrees of integration of those abilities within larger competencies. Consider an example common to didactic situations, where one uses a heuristic from one domain in another to facilitate learning, e.g., if one is asked to switch the hips in Brazilian Jiu-Jitsu with a back kick of the leg, but struggling until instructed, “like you are kicking your leg to propel yourself on a skateboard,” and suddenly, given the alternative frame, the ability is available. Here, the ability existed already in some genuine sense. And even though one could notice the affordance for a certain kind of backward kicking of the leg, it was not integrated into the larger competency network, and thus unavailable.

On the other hand, although the account of SFs suggests something about the rich topography of inter-regulatory dynamics characteristic of action, it is limited to a purely relational view of affordances, and how novel SFs (and their attendant affordances) emerge is not yet apparent. Moreover, Szokolszky et al. (2019, p. 17) write that “Enactivism has the … disadvantage of lacking an approach to perception that allows a coherent account of how organisms are connected/related to their surrounds.” But obviously such relations are assumed. The notion of structural coupling implicates the availability of enduring structures in the habitat. The ongoing reproduction of a particular umwelt (effectively a collection of autonomous sense frames) depends upon the ongoing availability of and connection with particular features of the habitat. Just as life requires the flow of certain biochemical structures for its reproduction, ways of life require the flow of certain sociomaterial structures too. Again, one can see here how the compatibilist approach is necessary. The abilities gearing an individual into a particular field of affordances are compatible with the networked structures characteristic of SFs, which are dependent upon the sociomaterial affordances in the form of life for their ongoing reproduction.

### Timescales

With the characterizations of the previous section in mind, the multiscale approaches of the enactive account and the SIF are mostly compatible. In both, any activity is always conceived as spilling over multiple scales simultaneously. Di Paolo et al. (2017, p. 147) write that “Habits do not stand in isolation as egotistically self-sustaining behavioral patterns. On the contrary, habits are nested in hierarchical, sequential, and ultimately networked relations in a kind of ecosystem …”. One such hierarchy is a temporal one. A simple “habit scheme,” such as *picking up the soap with your right hand*, is embedded in a larger “activity” (a habit network), *washing your hands after going to the toilet*, which is itself embedded in a “micro-identity” (a network of networks), *getting ready for bed*. As a general rule, we can see that activities that unfold at shorter timescales, such as short-lived sensorimotor coordinations on the timescale of milliseconds to seconds, are entrained (largely) to those at longer timescales, such as activities that unfold on the timescale of seconds to multiple seconds, and so on^8^. This provides conditions for adaptive responses at shorter timescales to accommodate the particularities of the situation while maintaining a course of action at the longer timescales. The organizational dynamics characteristic of each informs the normative dimensions of the unfolding situation.

In the SIF, when tending toward optimal grip, a compatible account is apparent. van Dijk and Rietveld (2018, p. 2) write that “when driving to a store, writing a text, or building a house, skilled individuals also adjust their activity in an anticipatory manner – people act adequately by anticipating situations as they unfold across larger scales, although often in a less certain manner than activities on smaller timescales.” These anticipatory dynamics depend upon action readiness patterns that are the consequence of being embedded in a “landscape of affordances” (e.g., Kiverstein and Rietveld, 2012). The concept of the landscape of affordances is intended to capture the multiscale entanglement of available affordances. As Bruineberg and Rietveld (2014, p. 3) put it, “The affordances of places (libraries, restaurants, etc.) typically constrain behavior over a longer timescale, while the affordances of objects nested in such a place, say the door to the library’s reading room, typically constrain behavior on a shorter timescale.” And so, when tending toward optimal grip, one is always mediating between the demands of multiple timescales.

There are some important shortcomings here, however, reinforcing the need for the compatibilist account. On one hand, without the autonomy of SFs, one cannot see how tensions emerge between timescales, something that is apparent in our experience, e.g., the tensions between one’s smoking habit and one’s identity as someone who lives a healthy lifestyle. Given that tending toward optimal grip pertains to the situation writ large, one might expect to be always achieving some sort of middle ground, but this is obviously not always the case. Sometimes, the norms of the smoking habit are satisfied in the fullest fashion possible, with one’s healthy identity providing a dissonant background. Given the autonomous dynamics of both SFs, it is clear to see how such relations manifest tensions. On the other hand, there are occasions where we experience solicitations over and above that which we have previously habituated at any timescale, possibly even as a resolution to the kinds of tensions just mentioned. This seems to be a consequence of tending toward optimal grip. Below it is suggested that this relation is central to the emergence of novel SFs.

### Environment

In *Sensorimotor Life* (Di Paolo et al., 2017), the notion of affordances is not developed in any technical sense. Nevertheless, they do speak about “the world” as “a constitutive part of any instance of sensorimotor coordination” (Di Paolo et al., 2017, p. 105), or about “dynamical mechanisms that allow environmental conditions to “call for,” or resonate with, certain sensorimotor schemes” (Di Paolo et al., 2017, p. 102). For Kirchhoff (2018), the lack of concrete enactive vocabulary concerning the “world” is a consequence of enaction’s focus on self-production, which aims at describing processes of system self-maintenance from within the system itself. As he puts it, within enaction, the “explanatory relation between living systems and the environment” takes “an internalist form, reducing the role of the environment in homeostasis” (Kirchhoff, 2018, p. 3). But, of course, this internalism is also a fundamental tenet of the enactivist perspective, for it highlights that the world “out there” is one about which our knowledge is enacted, and that we cannot but encounter it through our own individual histories of relating to it, even if we can do good science^9^. Given the centrality of this edict to the enactive position, the humble umwelt often appears the only environment of import and affordance but a handy term that affords description to certain aspects of experience.

The SIF, by centering the notion of tending toward optimal grip, places the regulatory load at the intersection of environment and embodied subject, suggesting that the developmental aim of the organism–environment system is to grow in synergy over time. Of course, this necessitates that there is something for the organism to synergize with. As Baggs (2018, p. 396) writes, “To understand the animal’s behavior … we must first understand what the animal’s behavior is directed toward … Having an account of structure in the environment is important because it provides a basis for understanding how an animal performs a particular task.” This ecological position reflects what Kirchhoff (2018) calls an “externalist causal–explanatory relation.” The externalist position is concerned with explaining self-preservation, which emphasizes the adaptive relationship between a changing environment and a changing organism. It builds upon Friston’s account of the FEP, which proposes that the “structural and functional organization” of a living system “is maintained by causal structure in the environment” and that “the hierarchical [statistical] structure of our brains is transcribed from causal [statistical] hierarchies in the environment” (Friston and Stephan, 2007, p. 418; taken from Kirchhoff, 2018).

Here, the habitat plays a much more explicit role than in the enactive account. In any FEP account, the “job” of the organism is to achieve and or maintain a “maximal fit between their probabilistic models and environmental niche *via* embodied activity,” sometimes referred as *active inference* (Friston et al., 2012; or more recently, *enactive inference* – see Ramstead et al., 2019). Basically, this suggests that organisms act in ways that minimize surprise (which is a measure of free energy) by actively taking part in their environment so as to produce sensory dynamics that align with what they anticipate to be the external causes of those dynamics. Such alignments are spoken about in the SIF in terms of tending toward an optimal grip. Thus, a kind of pervasive norm guiding the activity of the embodied subject is to progressively align with the structures of the habitat^10^.

Here again, the value in the compatibilist approach is apparent. Without a structured umwelt structure in the habitat means nothing, but without structure in the habitat, a structured umwelt cannot evolve or be sustained. Occupying one perspective and then the other is a little like switching between the different aspects of an optical illusion in which one can only really appreciate one or another image at a time, despite knowing that both are available to perception.

### Identity and Normativity

This section highlights some inconsistencies with the above accounts that turn on many of the distinctions already made. These will be central to the positive account that follows and further point to the necessity of a compatibilist approach.

Sense-making describes regulatory activities that support the ongoing individuation of autonomous organizations, be they autopoietic or habitual. However, understood as describing the self-*maintenance* of autonomous organizations, it runs into trouble, the core trouble in the relational account of affordances also. Namely, we do not just *maintain* existing structures, but bring about novel ones too. The sense-making that supports the maintenance of such organizations requires an existing “identity” to maintain, but it does not account for the emergence of such identities in the first place. Di Paolo (2020) does suggest at one point that sense-making is involved in the “construction” of “frames” (p. 36). But this is not the traditionally held position, nor is there presently any account that addresses this process in a way that overcomes the limitations elaborated herein.

The production of an identity is somewhat different than its reproduction. To apply a single term to both without significant qualification is not very helpful. Neither is it to apply a term initially proffered to explain the maintenance of life to the maintenance of ways of life. Similar points have been made elsewhere. Beaton (2014, p. 153) asks “how can non-sense ever become sense for us, if perception only ever presents the world within the existing structures of our understanding?” Or Weinbaum and Veitas (2017) write that enactivists “treat closure as an ideal point that delineates the existence of the individual in time, and … only from such a point and on sense-making is possible” (p. 382). The latter, looking to Simondon, shift their focus from the individual as their primary ontological category, in which the “genesis of individuals is merely the manner by which one individual transitions into another” and to the processes of individuation, what they describe as “the formation or becoming of individuals” (Weinbaum and Veitas, 2017, p. 376). In part II, I will suggest such a move is necessary when thinking about the coming into being of identities of the habit variety also^11^.

The recent *equilibration* account also recognizes something of this need. Di Paolo et al. (2017) synthesize an account of the “sensorimotor body” wherein sense-making takes on a broader characterization, more in line with the criticisms above, even if not explicitly. They suggest “Enactivism is concerned with explaining precisely these critical transitions between particular conditions that sometimes afford different functional descriptions and those ‘in-between’ dynamics that (re)constitute these or novel conditions” (Di Paolo et al., 2017, p. 27). However, when isolated, their account suffers. Di Paolo et al. (2017, p. 104) write that “Equilibration does not assume a “functional” source of normativity guiding adaptive change…” and wants to account for all change in terms of the “stability of individual schemes, along with their holistic coherence in the sensorimotor repertoire.” But tending towards optimal grip seems to reflect just such a source, and as will be observed below, when combined with the “stability of individual schemes” and their “holistic coherence,” can provide for an account of the individuation of novel habits in a way that avoids the limitations of existing approaches.

There is an oddity within the SIF also. The SIF borrows from Varela et al. and speaks about normativity, at least in part, as an upshot of identity preservation. For instance, they write that “Autonomous systems… have purposes of their own that arise out of the struggle to sustain their identity through the regulation of their coupling with the environment. They have an individuality and identity, and based on this identity, they are differentially sensitive to an environment of things that matter to them and are thus meaningful and valuable” (Kiverstein and Rietveld, 2018, p. 151). However, identity preservation here refers only to the biochemically individuated entity. Indeed, it does so despite their implications otherwise. They evoke the limitations spoken about earlier, suggesting that the normativity governing cognitive systems and that governing life are not straightforwardly equivalent. They also recognize that any living system, in the course of its life, will produce and sustain multiple identities. But despite momentarily recognising that such identities can include “patterns of sensorimotor behavior [that] can quite literally take on a life of their own,” they nevertheless reaffirm the position that they “interpret the enactivist concept of “identity” to refer to the biological organization of an individual that is maintained over time through material and energetic exchanges with the environment” (Kiverstein and Rietveld, 2018, p. 152). Moreover, it is hard to see where the former of these insights, relating to autonomous “patterns of sensorimotor behavior” are integrated into the SIF. Indeed, it seems they cannot be without recognizing that abilities are underpinned by autonomously organized habitual structures with their own self-generating norms.

The proposed solution to the above issues is to argue for a compatibilist approach. SFs supply the norms for the bulk of the self-maintenance, and it makes sense to think in such terms when thinking about the ongoing reproduction of the umwelt. But tending toward “an overall grip on the situation” (Kiverstein et al., 2019, p. 2859) can supply a more general norm when existing norms will not do. This tendency enables one to pick up information that supports the production of SFs, establishing novel interdependencies between bodily structures and structures in the habitat, transforming the umwelt in the process. In a compatibilist approach, situational demands and the demands of self-production are constantly being negotiated. In part II, such compatibilities support an account of the individuation of novel SFs and the relational affordances they embed.

## Part II: Enhabiting

Enhabiting provides an account of the individuation of sense-making frames based on the emergence of interdependencies between bodily structures and structures in the habitat. From a compatibilist perspective, it is the set of process by which features of the habitat of a species become incorporated into and transformed as features of the umwelt of a particular individual. This account of enhabiting takes inspiration from the Simondonian account of individuation.

### Simondon

Simondon’s philosophy of individuation takes on the question of becoming at the level of individual entities (physiochemical, biological, psychological, social). How do individuals both come into being, and maintain their being thereafter? How do the boundaries and distinctions that characterize the individual take hold without any individual preceding them? Simondon starts with the supposition that what is primary is not the individual but the processes of individuation. Any “individual” is something like a time slice of those processes. Writing about Simondon’s approach, Weinbaum and Veitas (2017, p. 377) suggest that, “For him, the individual is a metastable phase within a continuous process of transformation…” The “individual” then is an abstraction from the primary reality that entails ongoing processes of individuation. Giving some indication as to what this process might entail, Simondon (1992, p. 300) himself writes, “Individuation must… be thought of as a partial and relative resolution manifested in a system that contains latent potentials and harbors a certain incompatibility within itself…” Technical features of Simondon’s account have already been mentioned. Let me try to disambiguate the core features here before putting them to use.

The first feature is *metastability*. The term, as it is typically deployed today, comes from dynamical systems theory and describes systems that are relatively stable but not occupying any one particular deep well of attraction. Engstrøm and Kelso describe a metastable system as one in which “no stable or unstable fixed points remain, yet dynamical remnants of attractor∼repellors linger, giving rise to a dynamical flow…” (Engstrøm and Scott Kelso, 2008, p. 4). Simondon’s use reflects such a definition quite well, although it has its own emphasis. Combes (2012), describing Simondon’s use of the term, speaks about a physical system being “in metastable equilibrium… when the least modification to the parameters of the system (pressure, temperature, etc.) is sufficient to break the equilibrium of the system” (2012, p. 11). An example of a basic system in a metastable state is a wobbling bowling pin, which although kind of stable, might just as likely tip over as come back to standing, depending on the slightest change in conditions. Weinbaum and Veitas (2017) also offer the illustrative example of two people engaged in an argument. One can recognize from such examples a degree of tension is necessary for a system to maintain metastability. Indeed, any such system necessarily harbors potentials that are effectively incompatible. Metastability is ongoing if the system has not exhausted these potentials, e.g., the bowling pin has not come to rest, the argument has not died out.

A second feature of Simondon’s account is the notion of *intensive differences*. Intensive differences (or intensities) are effectively the drivers of individuation. They are “energetic differences that drive structural and state changes in a system” (Weinbaum and Veitas, 2017, p. 376). In the example of the argument, the intensities can include each interactant’s personal convictions. These concerns animate the metastable system, potentially leading to breakdown, but also potentially resulting in *consensual structure*. If they find a point of commonality, or if one is convinced by the other, there is a *determination* of a shared understanding, e.g., an agreed upon solution and a momentary relaxation of intensities. Weinbaum and Veitas write that these “intensities are correlated to the measure of metastability and level of structural changes taking place in the system. Low intensities are associated with relatively more stable dynamics, while high intensities are associated with volatile dynamics and swift structural changes” (Weinbaum and Veitas, 2017, p. 377/8). In other words, if there is no tension, there are no drivers of individuation present, and metastability is unlikely to emerge. Equally, if tensions are too severe, the determination of consensual structure is less likely, or will be much more dramatic. Most individuation proceeds within the sweet spot of low intensities. Think of the likelihood for the determination of some shared understanding in the context of a tiff as opposed to a bitter row.

Intensive differences arise within the context of a *problematic*. In the argument example, the problematic might be a work situation where the interactants need to coordinate on a project. Differing views on how best to approach it comprise the intensities that drive individuation, ultimately leading to some emergent consensual structure in the form of a shared plan. As Weinbaum and Veitas put it, “individuation of systems in general always starts from a situation of disparity. It takes place in the course of gradually establishing a coordinated exchange of signals among gradually differentiating elements that together bring forth a system” (Weinbaum and Veitas, 2017, p. 378). In this fashion, the system individuates and acquires an identity of its own, resulting from the coherence that has emerged between the involved agents. At any time, the system includes both consensual structure, comprising its previously individuated aspect, and ongoing intensities that drive future processes of individuation and either reproduce the previously achieved consensus or lead to its breakdown. These latent potentials, these unresolved intensities, Simondon refers to as the *pre-individual* elements in a system.

Any particular determination is highly dependent on its context and is in fact a codetermination between structural and behavioral aspects of the elements involved. The ongoing individuation of a persisting entity entails a trail of progressive determinations, a process referred to as *transduction*. For Simondon, this is a very general characterization and is taken to hold across domains, from the physiochemical to the social, in any of which it demands more specific description. However, there is a general logic at work here worth spelling out. The process of transduction describes a chain of operations on structures with each operation serving as a transformation of one structure into another, and every structure mediating between one operation and the next. Structure and behavior thus have a co-constraining effect: structure enabling the behavior that might follow from it and behavior enabling the (re)production of structural coherence. Transduction can start off quite messy and random, but as it progresses, invariants emerge such that “sets of structures and operations become mutually bounded,” and an “individuated entity arises which may either further consolidate or eventually disintegrate” (Weinbaum and Veitas, 2017, p. 379). Such entities, one might notice, have much in common with SFs, in which the organization enables behaviors that in turn enable the reproduction of the organization.

### Autonomy and the Pre-individual

Di Paolo has acknowledged the import of the Simondonian perspective for enaction, writing that it “makes explicit the material conditions of autonomy and introduces new elements for enactivism such as the notion of pre-individual criticality as inherent in the living body” (2016, p. 14). Integrating certain ideas from this account with its notions of autonomy, sense-making – ideas that are “only implicit in Simondon” (ibid.) – and tending toward optimal grip, I introduce the notion of *enhabiting*: a compatibilist account of the individuation of the novel SFs that comprise the umwelt, one that retains a strong appreciation for the role of habitat (as a source of pre-individual potential) in its production, reproduction, and transformation. As such, the notion of enhabiting is a metatheoretical concept. Straddling frameworks with different starting points, it invites us into a somewhat liminal space that is sensitive both to the umwelt and the habitat and focuses attention on the point at which the former is transformed within the latter. It, you might say, provides a metastable perspective from which to inquire into the dynamics of habituation and the emergence of relational affordances.

### Enhabiting Proper

This is the basic account. Situationally tending toward an optimal grip one is also sense-making at multiple timescales simultaneously and thus acting according to the self-generating norms of multiple relevant SFs. However, intensities can arise between existing SFs at various timescales and situational demands, manifesting tensions with no practiced path toward reduction. If the system does not simply break down or default to existing SFs but can be held as metastable in tending toward an optimal grip^12^, a momentary embrace of higher degrees of dissonance can provide an opening in which novel interdependencies can emerge between bodily structures and structures in the habitat, which thereafter form the basis for new SFs. This ongoing process in which novel SFs emerge (or existing ones are further consolidated) I refer to in terms of enhabiting. An example will be helpful.

#### Seeding a Habit

Enhabiting is ongoing all the time to varying degrees. There is, however, a scale of description that might offer a window onto these processes as they apply to our everyday experience. Before developing these examples however, it is important to prefigure them with the recognition that when one applies this as a lens through which to make sense of our everyday experience we are attempting to establish continuities between the domain of theoretical biology, in which these ideas can be more formally worked out, and into what is effectively the domain of folk psychology. Herein, the attempt is to develop accounts that help make intelligible the unfolding of everyday experience from the perspective of one who is concerned with such unfoldingings, and hopefully do so in a way that does justice to their unfolding within biochemically instantiated entities in reciprocal exchange with their environment. In this way, the following discussion is one in which assertions of and are always posited as if the rest of the world we able to be held stable, and as if our concepts might reliably map to mind independent features in the world. With these caveats in mind, some examples will be helpful.

The first example developed here is of you attempting to develop a consistent exercise routine. This type of example is chosen for some very specific reasons. Firstly, those actively and consciously engaged in the processes of behavioral change have recognized some basic regularities within the processes themselves, and some guiding principles that make the stabilization of novel trajectories of action more probable (e.g. Fogg, 2019). It so happens that they parallel the Simondonian accounts of individuation quite well. A couple of stereotypical examples of change efforts are outlined and compared. The differences in the efficacy of approaches can help illustrate the details of enhabiting as necessary. As will be observed, what might be recognized as the typically more successful approach better approximates the conditions laid out by Simondon as important to the individuation of novel structure.

Secondly, when actively pursuing a behavior change a kind of meta-normative dimension emerges that works as a kind of implicit problematic (to use Simondon’s term) coordinating the various components of the system under change. As such, at least for the purposes of an illustrative example, it offers a more circumscribed set of relevant processes that need to be included in the description, and thus a good starting point. Di Paolo has recently suggested that something that is missing from an enactive account is a “detailed look at the existential structure of becoming in conjunction with an operational/theoretical description of its relevant processes” (Di Paolo, 2020, p.3). What is provided here aims at precisely such an effort^13^.

You habitually display a set of activities that reflect a personal identity that might be named “healthy person.” Although you have not previously maintained a consistent exercise routine, you find yourself curious about the possibility, though tending to give yourself justifications for why you are not exploring it whenever the opportunity arises: you “haven’t got the right space,” the “right equipment,” the “time to get to a gym” etc. Then, you move into a new house in which your new housemate exercises regularly with some gym equipment in the basement and tells you that you are welcome to join. Now you are resourced with everything you would need to engage the practice. You decide to join her with the ardent commitment that you are going to take it very seriously, envisioning yourself a competent exerciser within no time. However, within a couple of days of exercising one hour per day, you find yourself making excuses, and within the week have fallen off entirely. Your experience is not one of optimal grip, but of wild deviations from optimal. But from what are you deviating from? What is producing the dissatunement that now calls for some action or set of actions to bring about its reduction, ultimately leading to the abandonment of the practice?

A necessary starting point is the recognition that herein multiple existing habituated SFs are giving rise to a host of norms shaping your activities across levels of organization and timescales. Of course, in reality, the ecology of relevant habitual structures will be impossible to define and disambiguate. Nevertheless, there are some reasonably clear invariant patterns that suggest a degree of autonomy that can be abstracted and used as lenses through which to discern the various normative dimensions of the situation. At shorter timescales, norms embedded with habits and habit schemes that pertain to the avoidance of pain, the navigation of the gym equipment and the coordination limbs, muscles and breathing patterns in novel ways; at longer timescales, norms embedded within micro-identities of being efficient in your actions so as to get to work on time and within personal identities that might be described as “efficient learner.” The experience of optimal grip in part relates to one’s actions being concordant with such norms across timescales. Another way of saying this is that one is satisfying the self-generated norms of the SFs presently enacted by acting within the boundaries of viability they supply. However, rarely are norms across all timescales perfectly synergistic within a given situation, and particularly in novel situations such as this. What is more common, and is the case here, are incompatibilities of varying degrees between situational demands and the self-generated norms of SFs at varying timescales.

Initially, your grip on the situation maintains a kind of optimality, for you are satisfying the various norms constraining your action: variables relating to the experience of pain are all within viability, sensorimotor the same, you have plenty of time before having to leave for work, and you appear to be successfully enacting your identity as an efficient learner. Such an experience is likely to generate a deep sense of being well located. However, before long, simply exercising – say, for instance, you have started out on a rowing machine – proves to be something of a chore, and deviations from optimal abound, limits of viability are breached, and the self-regulatory norms that aim at some prior homeorhesis now animate you. You feel pain in your back and something in the way you are bending your knee feels off, but you don’t appear to be able to negate such dissatunements regardless of your adjustments. The warm up program in the machine proves difficult to follow and you start to think that you are truly awful at rowing. All of this seems somehow incompatible with your identity as an efficient learner, and you start to question yourself. By day three you have bailed because you have “too much on at present to give it the time it deserves.”

In Simondonian inspired terms, the situation of committing to a practice one has not done before with the specific intention of bringing about a change represents a problematic (a task constraint that helps coordinate the components in the system), and the norms of existing SFs at various timescales (e.g. tendencies to avoid pain, identities as someone who gets to work on time and is an efficient learner) and the structures that support them comprise the intensities. These intensities are pregnant with preindividual potentials, and under this problematic can either lead to the breakdown of the system and its reorganization into some previous structure (as in the above example in which you abandon the practice), or, to the enhabiting of some novel SFs if the system can maintain metastability. In the example thus far, the former is a more apt description. Intensities are simply too pronounced, and thus the system defaults to some pre-existing habitual organization. Let’s compare this stereotypical example of a failed effort towards behavior change with an example guided by a core principle of successful behavior change as championed by B. J. Fogg, the founder of Stanford’s Behavior Design Lab. The principle is basically this, if you want to develop a new habit, make it small (Lieber, 2016; Al Marshedi et al., 2017; Fogg, 2019; Fogg and Euchner, 2019; Olt and Szasz, 2019). It’s important to say here that the “habit” that eventually emerges as the micro-identity that might be described in terms of one’s exercise routine, is not at all straightforwardly equivalent to habits as understood by Fogg. The equivalence is rather one in which a new trajectory or set of invariances in one’s action is opened up and stabilized. Fogg would likely refer to this as a habit. In the language developed here, however, this new trajectory reflects an multiscale ecology of inter-regulatory habits that acquire some degree of coherence and closure. The present account is thus not intended as advice on how to change behavior (though it may be informative to such an account), and Fogg’s work is leaned on only as an orienting device.

Imagine instead of taking the above approach, you commit to exercising for a couple of minutes every day for the first week, increasing each week thereafter for five minutes until you reach a practice time you are happy with. Besides the length of time you have allotted for practice, the same norms are operative. This time, however, norms embedded in habits relating to pain and discomfort are maintained mostly within viability, except for some slight tensions in your back; there is a newness to the sensorimotor coordinations the exercise demands, but nothing too strange; you have plenty of time before having to leave for work; and, you are acting from comfortably within your identity as an efficient learner. Under these constraints, although by two minutes you are experiencing some slight dissatunements and you have a distinct sense of being a “beginner,” it is nothing greatly outside of what you might have anticipated. Within a couple of days, the routine generates no feelings of dissatunement whatsoever, and a host of novel relational-affordances pertaining to the various aspects of the practice are available that previously weren’t. Moreover, encouraged by the experience, the practice begins to solicit as a general course of action, and you find yourself looking forward to the slight increase in time each week.

In this example, and, I might suggest, what undergirds the success and attendant popularity of the “tiny habits” approach, the problematic is one in which low intensities prevail. Relatively low intensities are, as previously noted, associated with more metastable dynamics, and so the experience of optimal grip can be somewhat retained even when not acting strictly according to the norms of existing SFs. In other words, although the norms of some existing SFs are deviated from, such deviations are slight enough that the system does not fall back into some previously sedimented SF. Here, an opening is found, one in which novel interdependencies between bodily and environmental structures have the opportunity to stabilize, enhabiting novel SFs with their own self-maintaining norms. Tending toward optimal grip, novel SFs have being enhabited that carry your activity through a particular course of action for a particular period of time. These allow you to make sense of the ongoings therein and adequately anticipate some set of contingencies likely to arise.

Enhabiting emphasizes a kind of transformation in which dispositional affordances in the habitat of a particular species enable the emergence of relational affordances in the umwelt of a particular individual. It is the initial mutual bounding of structure and operation resulting from action that transforms or consolidates existing habits, or leads to new ones; the ongoing sensorimotor (or affective, or linguistic) constitution of habit structures at multiple timescales, orchestrated by the tendency towards optimal grip. It is the process, from within a compatibilist perspective, by which an “individuated entity arises which may either further consolidate or eventually disintegrate” (Weinbaum and Veitas, 2017, p. 379). The Simondonian notion of determination most closely resembles the notion enhabiting as developed here. However, given the precariarity of SFs and their tendency to dissipate without reinforcement, enhabiting is intended to capture something of the notion of transduction also, in which a given structure can be more or less definitively individuated with successive determinations. Thus, we can speak about enhabiting in terms of degree, suggesting something about the degree of closure a given habit has acquired through repetition. In other words, SFs may start out as autonomous systems with poorly defined boundaries and so on, progressing towards greater degrees of autonomy with time and repetition, becoming more clearly articulated, more obdurate, and more trans-situational. Think of how the exercise habits of the beginner will be precarious, fragile, and dependent upon an ecology of supporting habits, whereas the habits of the longtime exerciser, who has personal-identities that have consolidated around such practices, will be much less dependent upon the enabling constraints of one particular environment (though they of course remain part of a larger ecology mediated by particular environmental structures). At such a level of organization an interesting dynamic is present, such that the endogenous side of the structural coupling begins to take some precedence. The traveling exerciser who carves out a space for their routine upon getting to a new room, and so on.

#### Hesitation and Symmetry Breaking

Although there is not adequate space to develop them properly, there are a couple of promising ideas that might help deepen an understanding of enhabiting. The first is the Bergsonian notion of *hesitation*, particularly as it has been revived within critical phenomenology (e.g., Al-Saji, 2014, 2018). Alia Al-Saji has been central in this effort, applying it to an understanding of interrupting racializing habits of perception. However, it can be applied more broadly too. In short, hesitation simply points to the “temporality and space required to interrupt habitual patterns of perception” (Dolezal and Petherbridge, 2017, p. 7). Precisely such an interruption is necessary if novel interdependencies between bodily and sociomaterial structures are to stabilize. Reflecting the dynamics of enhabiting articulated above, in which existing SFs are not adequate to the task, Al-Saji writes the following:

These are events for which we cannot account from within our instituted system of meaning – events that reveal, if we are open to them, the fractures in the coherence of the visual field. There are two ways of responding to such events: by maintaining the normative organization of the field and refusing to see them, or by receptively allowing an event to insinuate itself into our vision as the dimension according to which the visual field is restructured – thus changing how we see.

(2014, p. 155).

Although Al-Saji refers solely to the visual field here, there is no principled reason why this precise understanding may not be applied to the processes of habituation more generally. When we hesitate, we allow “the time both for a situation to be undergone and affectively registered and for marginal self-awareness, searching, and recollection to take place” (Al-Saji, 2014, p. 146). What results, according to Al-Saji, is an “opening,” which must be “*taken up* for new possibility to be created” (Al-Saji, 2014, p. 149). By maintaining a grip on the overall situation, by hesitating and resisting the overdetermination of the situation by falling back on existing SFs, we can “take up,” or “enhabit,” new relationships that reflect new routes, modes and patterns of being, becoming “responsive to what … [we have] … been unable to see” (Al-Saji, 2014, p. 147).

The second notion is the idea of *symmetry breaking*, which comes from the maths of pattern formation, and abstractly describes a process in which order emerges in physical systems. For mathematicians, the degree of symmetry in a system is the degree of invariance present in that system under transformation. The more transformations that can be made that leave it looking unchanged, so-called s*ymmetry operations*, the greater the symmetry (Ball, 2009, p. 20).

Consider a perfect sphere. The sphere can be rotated indefinitely upon its axis without variance. Moreover, reflections across its axis, in which one side is mirrored back upon the other, are also infinite. It has an infinite number of transformations without change under the operations specified here and thus has high symmetry. Contrast this with a five-sided star, which has only five rotations and five reflections across its axis, a total of 10 possible symmetry operations under transformations accounted for here. In the five-sided star, we observe more order than in the sphere, but this order, somewhat counterintuitively, is the result of a breaking of symmetries. Thus, the transition from uniformity to order can be thought to entail symmetry breaking. As Brender puts it, “the question of the genesis of form is not how symmetry arises out of disorder, but rather how the symmetry of disorder gets *broken* in determinate ways to produce the characteristic asymmetries of the forms we find in nature” (2012, p. 267).

Brender (2013) has tied these ideas to the notion of sense-making. Following Merleau-Ponty, who was wont to point out that it is the difference between figure and background that makes perception possible, Brender contends that it is the “asymmetry of the body’s environment that makes the perceptual regulation of movement possible” (2012, p. 240). The texture of such differences is precisely what allows for the getting of a perceptual “grip.” Such asymmetry, however, is also revealed by movement. Bodily movement helps reveal asymmetries as variation under transformation, the movement itself being the transformation which engenders variations in the perceptual field. Importantly, differentiation here is not a one-sided affair but is something that happens in the whole body–world relation. Combining these ideas with the notion of hesitation, one might suggest that hesitation provides the conditions for subtler forms of transformation, which in turn helps bring forth distinctions not previously available. If such distinctions support the general tendency toward optimal grip, structural interdependencies and new situational specific norms can be stabilized. The extent to which these ideas work well with the above account has not yet been adequately explored, but they seem promising.

### Environments in Enhabiting

A final point in need of emphasis is the role of the environment in enhabiting. Enhabiting novel SFs is a process of establishing interdependencies between structures in the habitat and structures in the body that thereafter support the maintenance of our ways of life. The enduring invariant structures that any habitat provides prior to being “internalized” in the process of enhabiting supply potentialities which when in contact with the perceiving subject limit that subject such that only some forms of relation are possible. Speaking about the role of environment in Simondon’s account of individuation, Mark Hensen writes the following:

… the upward spiral of individuation is driven … [in part by] … the coupling of individuation with the entire environment as a source of “preindividual,” “metastable” potential. [This helps]… ensure that emergence qua individuation involves a recursivity that is not driven solely or primarily by the organism’s demands but that instead draws from the global situation – the preindividual as potential – within which all individuations necessarily occur.

(Hensen, 2009, p. 134)^14^

This position supports the ecological claim that affordances are enduring structures in the environment that drive adaptation, and the attendant SIF claim that tending toward optimal grip progressively realizes an attunement between organism and environment. Moreover, as previously mentioned, Di Paolo has written that, Simondon “makes explicit the material conditions of autonomy and introduces new elements for enactivism such as the notion of pre-individual criticality as inherent in the living body” (Di Paolo, 2016b, p. 14). Here, one can see that these contributions do not simply relate to the biochemical resources supplied by our physical environments but also the sociomaterial resources supplied by our habitats. Enhabiting recognizes a process that extends beyond the embodied subject at its center and is in contact with the raw materiality of the world beyond. With our ways of life, we brush against the world and rub off it. The notion of enhabiting offers a bridging concept, a point of contact that can be acknowledged by both ecological and enactive approaches. To enhabit is to bring forth (to enact) within (to inhabit)^15^. We do not simply inhabit our worlds, we enhabit them, growing them in this or that direction according to the actions we take, reinforcing existing corners through revisiting them and letting the ones that no longer serve us die off due to our absence. Thus, it may be more accurate to speak of the habitual organizations that shape our umwelts at various timescales as “enhabitings”, emphasising their nature as active entities that animate our being in the world.

## Conclusion

The notion of enhabiting supports a dual-aspect view of phenomenal matter and can help deepen our sense of the compatibilities between ecological and enactive approaches in line with a radical embodied cognitive science. In doing so, it also provides a framing within which theories of learning from each approach, such as the enactive account of *equilibration*, or the ecological account of *direct perceptual learning*, can maintain productive conversations. Acknowledging such perspectivalism entails something of a metatheoretical move. In the philosophy of science, such moves are not without precedent. For instance, Roy Baskar’s philosophy of critical realism advances a so-called four planar theory in which any aspect of reality is to be understood as constituted by “four dialectically interdependent planes: of material transactions with nature, inter-personal action, social relations, and intra-subjectivity” (Archer et al., 2013, p. 566). In Bhaskar’s view, any of the planes can serve as a lens through which to make observations and conceptual distinctions, but any such lens is always in conversation with the others too, and any explanation given only in terms of one or another is only ever partial. I am not positioned to either endorse or reject Baskar’s view, I simply point to it as a precedent for the kind of move attempted here. However, given the epistemic limits set by enaction, the umwelt does hold something of a privileged position. This should not be taken as a stain on our abilities to do good science, but rather an injunction against excessive hubris. Herein I have attempted to make sense of the frames through which we make sense. This is an odd task. At times like this one does well to remember that the map is not the territory (Korzybski, 1933), neither two compatible ones!

## Author Contributions

The author confirms being the sole contributor of this work and has approved it for publication.

## Conflict of Interest

The author declares that the research was conducted in the absence of any commercial or financial relationships that could be construed as a potential conflict of interest.

## Footnotes

1 Baggs and Chemero do speak, at different points, of the “complementarity” of these approaches and their “unification.” Thus, it is not clear whether the compatibilist account developed here is perfectly aligned with their ambitions or not. Nevertheless, their account does provide a stable mooring from which a compatibilist account might venture.

2 The language of “externalities” comes from economic theory and pertains to the cost of an action on a third party who did not choose to incur it. Pollution is the customary example. It is used metaphorically here as a means to speak about what is “left out” or negatively affected by adoption of one particular framework or another. For instance, in the context of “mental” healthcare, the externalities of a reductive framework might be a failure to address underlying social conditions of disorder (see Alexander, 2010). The perpetrator is typically in denial of such costs and would most likely prefer that they were not incurred. It is simply presumed here that any theoretical framework will have some externalities, and thus they demand sensitive application.

3 See Kiverstein and Wheeler (2012) for an edited collection on the influence of Heidegger on contemporary cognitive science. Or Kaufer and Chemero (2015) for a more concise account, alongside the account of phenomenology more generally.

4 One might refer to such limitations as “theoretical externalities.” See footnote 2 above.

5 See Cummins, 2020 for an extended discussion of these particular relations.

6 See Flament-Fultot (2016) for a constraints-based approach that avoids the language of modeling but can still provide principled accounts of anticipation. Fultot makes the case for how a particular context “pre-constrains” the living system, resulting in an “anticipatory poise” relevant to acting therein.

7 I extend this language to the social too, wherein I speak about *participatory sense-making frames* (see James, 2020, for discussion).

8 Of course, there are numerous ways one can conceive of how the timescales of action should be carved up. See Loaiza et al. (2020) for a comprehensive account well aligned with the perspective developed here, but with a different set of heuristics, a “specification hierarchy” (Salthe, 1991), as opposed to the scalar hierarchy adopted here.

9 See Cummins, 2020, for a compelling presentation of the inescapability of this epistemic framing.

10 Any environment will, of course, be rich enough that there will be many well-aligned structures.

11 In recognizing this limitation, that “we tend to treat bodies more or less as givens, as starting points …” (2019, p. 2), Di Paolo has also recently aligned the Simondonian perspective with enaction. However, his efforts are conducted in a purely enactivist manner and take a different line than the one taken here. This emerging engagement with Simondon is a promising one for embodied cognitive science and, given the richness and originality of Simondon’s thought, is sure to be productive.

12 The notion of tending towards optimal grip may strike one as something of a deus ex machina in this context, attempting to explain the resolution of tensions into novel organization with something of a poorly specified “mechanism.” I have some sympathy with this concern, but for now I will simply say this. It is not entirely clear to me at this point how well enactive accounts focused on autonomy and accounts that lean on the FEP ultimately play together, but that our experience reflects a general tendency towards optimal grip over and above the normative dimensions of our existing habits does seem apparent. Thus, the notion of tending towards optimal grip at the very least, has some heuristic value. That said, my feeling is that the tension between these concepts reflects a larger theoretical tension between the theories within which these concepts are typically embedded, i.e. between accounts that center operational closure and those that center thermodynamic openness. Enactive approaches, as we know, tend to build their ideas with notions of operational closure center of mind (even if acknowledging the necessity of thermodynamic openness, e.g. Di Paolo et al., 2017, p. 115), more ecological leaning accounts tend to center notions of thermodynamic openness, even if sometimes acknowledging the role of operational closure (e.g. Chemero, 2009, Bruineberg and Rietveld, 2014; Rietveld and Kiverstein, 2014). In accounting for the individuation of novel habitual organizations, it seems one cannot, ultimately, igone either perspective. The notion of tending towards optimal grip then, is used here as something of a placeholder, reflecting the dimensions of thermodynamic openness relevant to the individuation of novel habitual forms, but not yet well developed in the relevant literature so as to constitute a “mechanism.” What this mechanism must ultimately account for is the means by which the system can maintain a kind of situational metastability, very much along the lines of the Simondonian account, that enables the resolution of tensions into novel forms. There is ongoing work that explicitly takes itself to be working in the generative space between these two theoretical positions (i.e. operational closure and thermodynamic openness), e.g. Mont́evil and Mossio (2015), Woermann (2016), Loaiza et al. (2020). Although there is not time or space to develop the relevant histories herein, or work a sophisticated understanding of the insights of such positions into the present account, the compatibilist account under development here can be taken to be allied with such positions. Beyond this thesis, the thinking developed herein will pursue such generative tensions and their relevance for a compatibilist cognitive science more wholeheartedly.

13 Such examples reflect an understanding derived from number of autoethnographic efforts (both successful and unsuccessful), familiarity with both popular and academic literatures pertaining to behavior change and frameworks for change, and my own developments towards a systematized practice for behavior change I refer to as Ecobehavioral Design (James, 2018). However, in this context these examples are not intended to be anything other than illustrative of the individuation of novel habitual organizations at multiple timescales, wherein, inspired by the Simondonian account of individuation, the dynamics that more reliably support the emergence of novel invariant patterns in individual behavior can be made intelligible through the compatibilist understanding being developed.

14 Text in brackets is my addition.

15 I am aware that the suffix “en” does not capture the meaning of “bringing forth.” “Enhabit” is simply a portmanteau of enact and inhabit. Elsewhere, I speak of “coenhabiting,” extending these ideas to the social domain also (see James and Loaiza, 2020).
