# Corrigendum: Bringing forth within: Enhabiting at the intersection between enaction and ecological psychology

**DOI:** 10.3389/fpsyg.2023.1141273

**Published:** 2023-03-03

**Authors:** Mark M. James

**Affiliations:** School of Computer Science, University College Dublin, Dublin, Ireland

**Keywords:** enaction, ecological psychology, sense-making, umwelt, enhabiting, Simondon, individuation

In the published article, there was a mistake in the **Abstract** as published. The **Abstract** previously stated, “In part two these compatibilities are brought together with the that these compatibilities can be brought together with the philosophy of Gilbert Simondon to develop the notion of enhabiting.” This should be, “In part two these compatibilities are brought together with the philosophy of Gilbert Simondon to develop the notion of enhabiting.” The corrected abstract appears below:

Baggs and Chemero (2018) propose that certain tensions between enaction and ecological psychology arise due different interpretations about what is meant by the “environment.” In the enactive approach the emphasis is on the umwelt, which describes the environment as the “meaningful, lived surroundings of a given individual.” The ecological approach, on the other hand, emphasises what they refer to as the habitat “the environment as a set of resources for a typical, or ideal, member of a species.” By making this distinction, these authors claim they are able to retain the best of both the ecological and the enactive approaches. Herein I propose an account of the individuation of habits that straddles this distinction, what I call a compatabilist account. This is done in two parts. The first part teases out a host of compatibilities that exist between the enactive account as developed by Di Paolo et al. (2017) and the skilled intentionality framework as developed by Bruineberg and Rietveld (2014) and Rietveld and Kiverstein (2014). In part two these compatibilities are brought together with the philosophy of Gilbert Simondon to develop the notion of enhabiting. Enhabiting describes a set of ongoing processes by which an umwelt emerges from and is reproduced within the relationship between an embodied subject and their habitat. Thus, enhabiting points toward a point of intersection between enaction and ecological psychology. To enhabit is bring forth (to enact), within (to inhabit).

The author apologizes for this error and states that this does not change the scientific conclusions of the article in any way. The original article has been updated.
